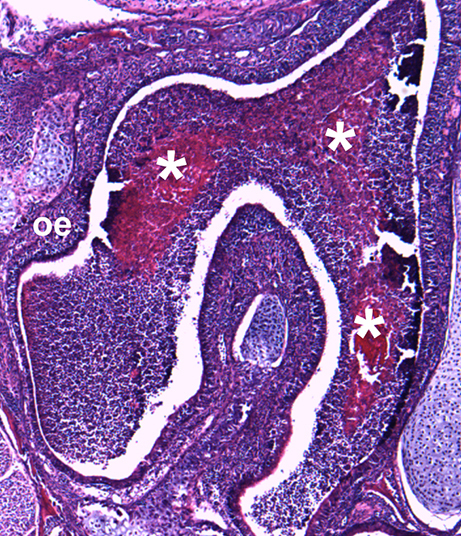# New insights into paediatric dysphagia from mice

**Published:** 2014-02

Up to 80% of children with developmental disorders can be affected by paediatric dysphagia – compromised ingestion, chewing or swallowing of food. This condition can have a severe effect on an individual’s quality of life and, left untreated, the disorder can cause food aspiration, choking or life-threatening respiratory infections. Despite its high co-incidence with developmental disorders, little is known about paediatric dysphagia, in part because of the lack of animal models that mimic dysphagic symptoms. In this study, Anthony LaMantia and colleagues report that features of paediatric dysphagia emerge during early postnatal life in a mouse model of DiGeorge (22q11.2) deletion syndrome (22q11DS), a common developmental disorder. They demonstrate that the symptoms are associated with disrupted development of cranial nerves that are important for feeding and swallowing. These defects are preceded by alterations in the expression and patterning of specific transcriptional regulators, particularly retinoic acid (RA)-sensitive genes. Importantly, the defects can be rescued by diminished RA signalling. These findings demonstrate that the 22q11DS mouse model could be a powerful tool for dissection of the neural circuitry involved in paediatric dysphagia, and could be used to pinpoint therapeutic targets. Page 245

**Figure:**